# Socio-economic predictors of adolescent marriage and maternity in West and Central Africa between 1986 and 2017

**DOI:** 10.7189/jogh.11.13002

**Published:** 2021-08-10

**Authors:** Vera Sagalova, Jonathan Garcia, Aline Simen Kapeu, John Ntambi, Noel Marie Zagre, Sebastian Vollmer

**Affiliations:** 1 Heidelberg Institute of Global Health, University of Heidelberg, Heidelberg, Germany; 2 Department of Economics and Centre for Modern Indian Studies, University of Goettingen, Göttingen, Germany; 3 UNICEF, Regional Office for West and Central Africa, Dakar, Senegal; 4 UNICEF Area Representative for Gabon and São Tomé and Príncipe and to the ECCAS, Libreville, Gabon

## Abstract

**Background:**

Early marriage and maternity prevalence rates among adolescent girls remain alarmingly high in West and Central Africa (WCA). This study aims to explore the associations between socio-economic factors and the prevalence of early marriage and maternity, thus contributing to the identification of girls at risk of early pregnancy or marriage.

**Methods:**

We pooled data from national representative surveys (1986 – 2017) for 23 countries in WCA to examine associations between wealth, educational attainment, religious affiliation, and place of residence with adolescent marriage and maternity. We decomposed the wealth and education gradients for individual countries, while controlling for common characteristics of the local environment via the use of primary sampling unit fixed-effects. The pooled sample provides information on 262 721 girls (age 15-19 years). Survey weights and population share weights were used in the estimations.

**Results:**

The prevalence of adolescent maternity and marriage exhibited a wealth and education gradient. Prevalence of marriage in the poorest wealth quintile was 41.1% (95% confidence interval (CI) = 38.8%-43.5%) and 10.5% (95% CI = 9.5%-11.6%) in the richest. For maternity it was 38.3% (95% CI = 36.4%-40.3%) in the poorest quintile and 12.7% (95% CI = 11.5%-13.9%) in the richest. Marriage/maternity is three/two times more likely to occur among girls with incomplete primary or no formal education than in those with at least primary. Maternity and marriage among adolescents exhibit a geographical pattern and differences between religious groups. Adolescent marriage prevalence was 34.4% (95% CI = 32.9%-35.8%) in rural areas compared to 13.3% (95% CI = 12.3%-14.2%) in urban areas. Adolescent maternity prevalence was 32.8% (95% CI = 31.7%-33.9%) in rural compared to 16.3% (95% CI = 15.3%-17.3%) in urban areas. Finally, the prevalence of adolescent marriage was substantially higher among Muslims compared to all other religious groups.

**Conclusions:**

Our results highlight the disparities in the prevalence of adolescent marriage and maternity and confirm the existence of wealth and education gradients. These findings can help to improve targeting of vulnerable adolescents and to identify areas for policy implementation.

Giving birth at an early age has long-lasting consequences for young women and their children. A vast body of literature has documented the negative consequences of early marriage and maternity on mothers’ educational and economic opportunities, as well as on their and their children’s health [1-3]. In the developing world, this affects 19% of the adolescent female population (girls aged 10 to 19) who become pregnant [4]. Furthermore, early motherhood is associated with early marriage in the sense that nine out of ten births to adolescents in low-income settings happen in the context of a marriage [5]. Adolescent marriage constitutes a human right violation, regardless of gender [6], however, young girls are affected disproportionally [7,8]. Global estimates propose that one in three women enters a union before the age of 15 [9], while for men this is the case for one in twenty one [10].

Global efforts and initiatives such as Every Woman Every Child have contributed to the significant reduction in prevalence rates over recent years in both early maternity and marriage [11]. They also helped to recognize the central role of adolescents for the success of the 2030 Agenda for Sustainable Development in the updated Global Strategy for Women’s, Children’s and Adolescents’ Health 2016-2030 [12]. Despite these important achievements, progress has been uneven, resulting in the widening of disparities across regions and countries [5,13].

The West and Central African region (WCA, UNICEF geographic definition) has been characterized by a late onset in the reduction of maternity and marriage rates among adolescents and is currently the region with some of the highest prevalence rates worldwide [14,15]. In addition to disparities between geographic regions, empirical evidence suggests that cross-country differences within these regions are broadening due to country-specific characteristics such as income level or education system and due to varying speed in their catching-up process, generally resulting in a higher prevalence in the poorest and least educated countries [3,5,16]. West and Central Africa illustrates the severity of this issue while showing a prevalence of adolescent maternity that ranges from a high of 49% in the Central African Republic to a low of 16% in Senegal, and early marriage rates that range from a maximum of 61% in Niger to a minimum of 6% in Ghana [17].

Nonetheless, heterogeneity in the individual risk of early marriage and maternity is clearly extremely high within countries [18]: prevalence rates of maternity and marriage among adolescents often follows a geographical pattern within countries, reflecting the local and individual level variation of economic development and cultural practices [19,20]. Several factors might play a crucial role in the likelihood of early marriage and childbirth; Education, as certainly one of the most important mediators, is expected to delay marriage and childbearing by preparing girls for jobs and livelihoods, raising their self-esteem and their status and agency in their households and communities [5,21,22]. Similarly, increases in income and urbanization are expected to reduce these risks, while in contrast, cultural aspects such as religious affiliation might increase them by encouraging early marriage in an attempt to reduce the occurrence of premarital sex among girls [23].

This pathway has been studied and confirmed extensively: a large body of empirical literature has found that girls with higher educational attainment, living in urban settings, or belonging to a household in upper wealth quintiles face a comparatively lower risk of getting married or giving birth at an early age [4,5]. A meta-analysis of studies from 24 African countries found marriage, rural residence, not attending school, low parental education, and lack of communication on sexual and reproductive health (SRH) to be positively correlated with adolescent pregnancy [24]. Likewise, in Sub-Saharan Africa, poverty, marriage, religion, gender norms, access to contraceptives and affordable education are associated with adolescent marriage and pregnancy [25,26]. Contrarily, a lower prevalence of child marriage and teenage childbearing is observed in countries with laws that define (and enforce) a minimum legal age for marriage of 18 or higher [27]. In WCA region, single-country studies from Ghana [23], Nigeria, Burkina Faso, and Niger [28], as well as a pooled analysis of 15 countries [29] found similar patterns to those observed in the entire region of Sub-Saharan Africa.

In the present study we examine the associations between wealth, educational attainment, religious affiliation, and place of residence with adolescent marriage and maternity in 23 West and Central African countries. In addition to a pooled analysis for the entire region, we further decompose the wealth and education gradients for individual countries, while controlling for common characteristics of the local environment, such as cultural norms or average living standards. This study contributes to existing evidence base by identifying characteristics that are associated with an increased risks of becoming pregnant or married during adolescence and by exploring the heterogeneity between countries within this region in order to facilitate policy measures to improve maternal and adolescent programming in the West and Central African region.

## METHODS

### Data sources

We pooled all Demographic and Health Surveys (DHS) and Multi-Indicator Cluster Surveys (MICS) for 23 West and Central African countries that were conducted between 1986 and 2017 (no data are available for Cape Verde, a detailed list of countries and survey years for each outcome is provided in Table S1 in the **Online Supplementary Document**). The pooled sample provides data on 262 721 adolescent girls between ages 15 and 19. While some few younger adolescents are included in the pooled sample (877), they are excluded from present analyses as all observations stem from two MICS surveys (Equatorial Guinea and Guinea Bissau 2000) and are generally not part of the MICS or DHS sampling design.

### Outcomes

Outcome variables are indicator variables for marriage and maternity. The marriage variable is coded as one if the adolescent girl was ever married. The maternity variable is coded as one if she has ever given birth or is currently pregnant. A weighted average of these binary variables aggregated on any geographical entity can hence be interpreted as prevalence in this entity.

### Exposure

Exposures are education, wealth, rural/urban residence, and religion. The education variable has the categories “none or less than primary”, “primary or incomplete secondary” and “secondary or higher”. In regression analyses, the category “none or less than primary” serves as reference category. Wealth is measured through asset index quintiles. The poorest quintile serves as reference category in regressions. Rural/urban residence is expressed by a dummy variable for urban location, thus rural location being the reference group. We include religious affiliation as control variable because we expect that there is some variation in marriage practices across different religious groups. The variable is coded in four broad categories which are “Muslim”, “Christian”, “Traditional/Animist”, and “none or other”. Muslim religion is used as reference category in regressions.

### Statistical analysis

Descriptive statistics were calculated using survey weights for individual surveys and in the pooled sample each country was additionally weighted with its population share. All regressions were linear probability models. Some specifications include fixed effects at the primary sampling unit level to control for common characteristics of the local environment such as cultural norms or average living standards. In settings with large numbers of fixed effects, linear probability models are more robust than logit or probit models. All analyses as well as sample generation have been performed using STATA 14 statistical software package (StataCorp, College Station, TX, USA).

## RESULTS

**Figure 1:**
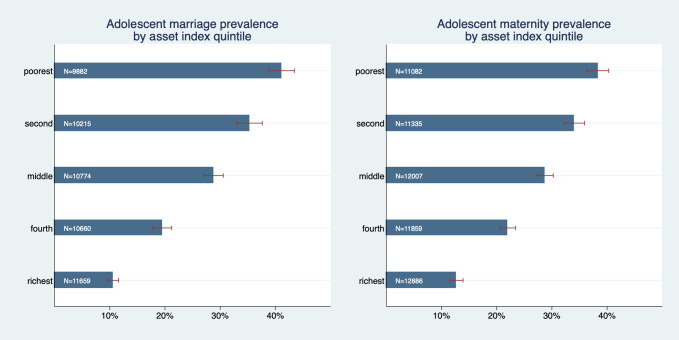
Adolescent marriage and maternity prevalence by asset index quintile.

**Figure 1** graphs early marriage and maternitiy prevalence rates by wealth quintile and exposes a strong wealth gradient for both adolescent marriage and maternity. In both outcomes prevalence was almost four times higher in the poorest quintile than in the richest quintile; Prevalence of adolescent marriage was 41.1% (95% CI = 38.8%-43.5%), in the poorest quintile, and 10.5% (95% confidence interval (CI) = 9.5%-11.6%) in the richest. Prevalence of adolescent maternity was 38.3% (95% CI = 36.4%-40.3%) in the poorest quintile and 12.7% (95% CI = 11.5%-13.9%) in the richest. The graph exposes a pronounced wealth gradient: differences between all quintiles are relatively large and none of the confidence intervals overlap.

**Figure 2:**
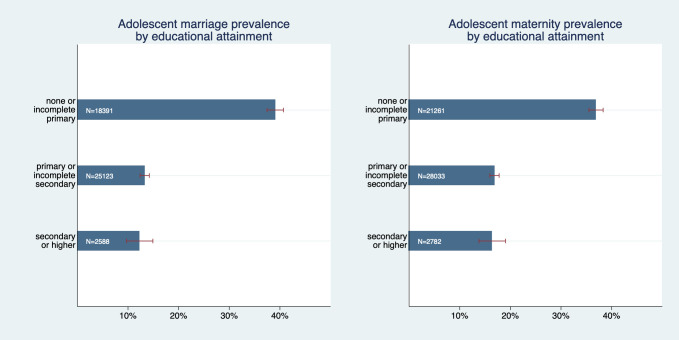
Adolescent marriage and maternity prevalence by educational attainment.

Moreover, we found a strong wealth gap between the lowest and all other educational categories (**Figure 2**); Marriage was roughly three and maternity roughly two times more likely to occur among adolescent girls with no or incomplete primary education as compared to the other two categories. However, prevalence rates of adolescent marriage and maternity cannot be statistically distinguished between the highest two educational groups. Adolescent marriage prevalence was 39.1% (95% CI = 37.4%-40.7%) in the lowest education category and 12.3% (95% CI = 9.7%-14.9%) in the highest. Adolescent maternity prevalence was 36.9% (95% CI = 35.5%-38.3%) in the lowest education category and 16.4% (95% CI = 13.8%-19.0%) in the highest.

**Figure 3:**
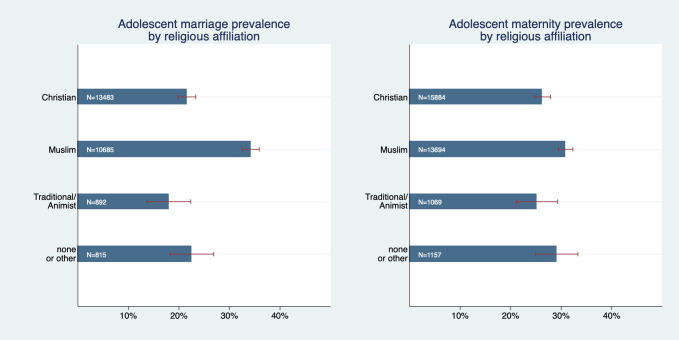
Adolescent marriage and maternity prevalence by religious affiliation.

**Figure 4:**
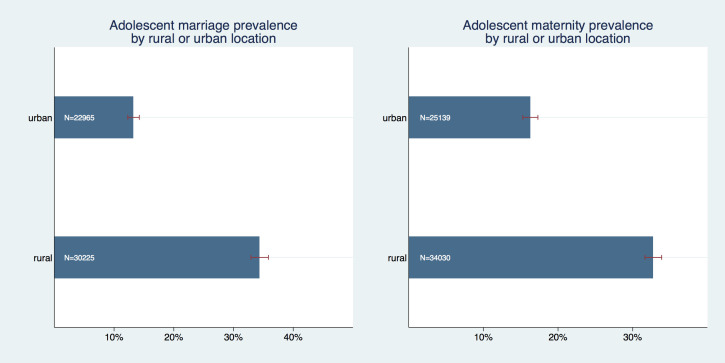
Adolescent marriage and maternity prevalence by urban or rural location.

We have also graphically confirmed that there are differences in early marriage and maternity between various religious groups (**Figure 3**); Prevalence of adolescent marriage was substantially higher among Muslims compared to all other groups with a prevalence of 34.2% (95% CI = 32.5%-35.9%). Adolescent maternity prevalence was also highest among Muslims, but here the differences to other religious groups were much smaller (within the magnitude of roughly 3-6 percentage points) and either not or only marginally significant. Both adolescent marriage and maternity prevalence were significantly higher in rural than in urban areas (**Figure 4**); Adolescent marriage prevalence was 34.4% (95% CI = 32.9%-35.8%) in rural areas compared to 13.3% (95% CI = 12.3% - 14.2%) in urban areas. Adolescent maternity prevalence was 32.8% (95% CI = 31.7%-33.9%) in rural areas compared to 16.3% (95% CI = 15.3%-17.3%) in urban areas.

These patterns were confirmed in regression analyses in which all factors were simultaneously controlled for, c.f. specifications (1) and (3) in **Table 1**. The coefficients only marginally changed when the models were adjusted for primary sampling unit fixed effects, which control for common characteristics of the local environment as well as implicitly time fixed effects (as PSUs are survey-specific), c.f. specifications (2) and (4) in **Table 1**.

**Table 1:** Regression results for outcomes “adolescent maternity” and “adolescent marriage” with and without inclusion of PSU FE*

	(1)	(2)	(3)	(4)
	**Adolescent maternity**	**Adolescent maternity (with PSU FE)**	**Adolescent marriage**	**Adolescent marriage (with PSU FE)**
Education:				
Primary or incomplete secondary	-0.140§ (-54.29)	-0.141§ (-54.09)	-0.211§ (-69.20)	-0.212§ (-70.33)
Secondary or higher	-0.119§ (-15.92)	-0.132§ (-18.59)	-0.213§ (-39.13)	-0.208§ (-37.27)
Wealth:				
Second quintile	-0.0112† (-2.50)	-0.0126‡ (-2.85)	-0.0202§ (-4.07)	-0.0241§ (-4.85)
Middle quintile	-0.0426§ (-8.88)	-0.0459§ (-9.77)	-0.0573§ (-11.51)	-0.0706§ (-14.40)

Fourth quintile	-0.0604§ (-12.32)	-0.0713§ (-15.55)	-0.0687§ (-13.32)	-0.0995§ (-20.11)
Richest quintile	-0.113§ (-19.92)	-0.135§ (-28.17)	-0.104§ (-19.25)	-0.153§ (-32.32)
Location:				
Urban	-0.0220§ (-5.51)		-0.0624§ (-16.03)	
Religion:				
Christian	-0.00274 (-0.82)	-0.00139 (-0.41)	-0.0871§ (-27.30)	-0.0910§ (-28.05)
Traditional	-0.0780§ (-9.54)	-0.0705§ (-8.53)	-0.160§ (-20.47)	-0.150§ (-19.25)
None or other	-0.0105 (-1.60)	-0.00642 (-0.98)	-0.111§ (-17.03)	-0.113§ (-17.40)
Constant	0.415§ (113.10)	0.413§ (125.96)	0.500§ (114.09)	0.494§ (127.54)
Observations	154 783	154 783	137 016	137 016

PSU FE – primary sampling unit fixed effect

*t statistics in parentheses.

^†^*P* < 0.05.

‡*P* < 0.01.

§*P* < 0.001

**Figure 5:**
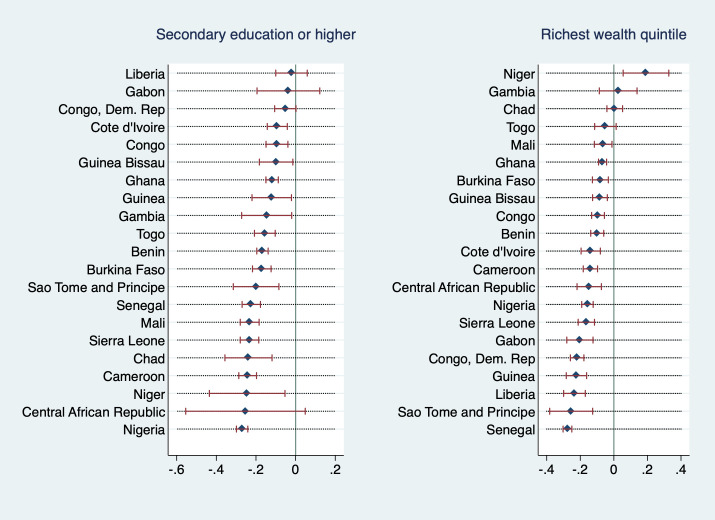
Regression coefficients (outcome: adolescent marriage) from adjusted model with PSU FE, by country (PSU FE – primary sampling unit fixed effect).

**Figure 6:**
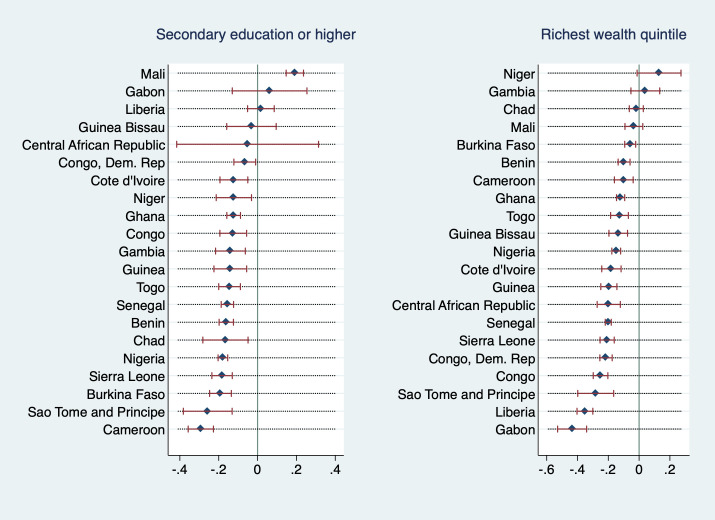
Regression coefficients (outcome: adolescent maternity) from adjusted model with PSU FE, by country (PSU FE – primary sampling unit fixed effect).

**Figure 5** and **Figure 6** explore the heterogeneity in the association of wealth and education with the outcome variables between countries. They display regression coefficients of the highest education and wealth category for each country from models that were adjusted for all other covariates, primary sampling unit fixed effects, and where the lowest education and wealth categories are the respective reference groups (analogous to model specifications (2) and (4), however on individual country level). As Equatorial Guinea and Mauritania are missing information on religion and hence cannot be estimated with the same model as all other countries in the sample, these two countries are excluded from these analyses.

Most coefficients were negative and statistically significant, some negative but insignificant, a few positive and insignificant, and two (education coefficient in the maternity regression in Mali as well as highest wealth quintile coefficient in the marriage regression in Niger) positive and significant. In general, only coefficients that are either positive or very close to zero were insignificant, with one exception: education coefficients were insignificant for Central African Republic, regardless of the measured effect size (which was second-highest in the sample). For adolescent marriage, education coefficients had negative values between roughly zero and -0.3 in all countries but were statistically insignificant for the Central African Republic, Congo, Dem. Rep., Gabon, and Liberia. Wealth coefficients were statistically insignificant for Togo, Chad, Congo, and Gambia with Chad’s and Gambia’s coefficients being positive (however, almost indistinguishable from zero). For adolescent maternity, education coefficients were statistically insignificant for the Central African Republic, Guinea Bissau, Liberia, and Gabon, in Mali they were positive and significant. Wealth coefficients were statistically insignificant in Mali, Chad, Gambia, and Niger (with coefficient for the Gambia and Niger being positive). In other words, for these countries, we cannot statistically distinguish the adolescent marriage and maternity outcomes for the highest and lowest wealth or education categories.

## DISCUSSION

This study sought to shed light on the association of adolescent marriage and maternity with select socio-economic characteristics in the entire West and Central African region as well as at the country level within this region. Our results confirm that individual-level characteristics are indeed associated with prevalence rates of adolescent marriage and maternity in this region. These findings are in line with relevant literature on the global and Sub-Saharan African scale [24-26,28]. However, we find heterogenous associations on the country level, where some wealth and education coefficients were insignificant and hence we cannot make any claims on the direction of association between the outcomes adolescent maternity and marriage and their predictors wealth and education in these countries. Moreover, in Mali we find that having secondary or higher education is associated with a higher probability of adolescent maternity by 19% (95% CI = 15%-24%) and in Niger we find a positive association between belonging to the wealthiest segment of the society and adolescent marriage. The underlying data does not allow to disentangle the driving mechanisms behind this paradoxical association.

In the context of the WCA region, we find poverty (expressed as belonging to the poorest wealth quintile in the present work) among the leading predictors of adolescent marriage and maternity, which is in line with existing empirical evidence [23,25,26,28,30].

Educational attainment emerged as an important determinant of the prevalence of adolescent marriage and childbirth. We found young girls who have completed at least primary education to be at a significantly lower risk of premature marriage and maternity than their peers without a formal education. This finding suggests that even low-grade education has a protective effect against early marriage and childbirth. In this context reaching literacy might be understood as one important threshold for protective properties of education, and in fact we explore this variable and find a maternity prevalence of 40.5% among illiterate (95% CI = 39%-42%) and only 15% among literate adolescents (95% CI = 14%-16%). The adolescent marriage prevalence is 45% among illiterate (95% CI = 44%-47%) and 12% among iterate adolescents (95% CI = 12%-13%).

Adolescent marriage represents one of the many barriers that prevent girls from attending school [31], but at the same time school attendance delays family formation decisions for girls. Thus, the relationship between adolescent marriage and education is bidirectional [27].

Rural areas exhibit a higher prevalence of early marriage and maternity than urban areas. Poverty, limited access to education, contraceptives, and services of sexual and reproductive health are believed to be important determinants of high prevalence rates observed in rural areas. [24,32]

In addition, we find religious affiliation to be associated with prevalence of adolescent marriage and maternity, and here young girls belonging to the Muslim religious group are disproportionally affected by both. In this context, it is important to stress that existing research suggests that it is not the religious belief in itself that perpetuates these harmful practices, but more likely the deep-rooted socio-cultural norms which are embedded in certain religious communities [33,34].

There are some important limitations to this study; Given the nature of our data, the findings are merely observational in nature and we cannot claim to identify any causation. This has been considered while interpreting the findings above. Nevertheless, the results are useful for the exploration of factors that increase young girls’ exposure to early marriage and maternity, as well as for identifying particularly vulnerable subgroups. An additional drawback is that our study focuses solely on (older adolescent) girls as young boys or younger adolescent girls are systematically left out from large standardized household surveys such as DHS or MICS amd hence no regionally representative data are available on these subgroups.

## CONCLUSION

The present study identified determinants of adolescent marriage and maternity in West and Central Africa as a whole and investigated differential patterns associated with girls’ educational attainment, wealth, area of residence, and religious affiliation across various countries in this region. We established that early marriage and maternity occur disproportionally within low educated and poor women in the region, but moreover in those belonging to Muslim faith. In addition, young women living in rural areas face a higher probability of becoming pregnant or getting married prematurely.

## Additional material


Online Supplementary Document
