# Supplementary material for: Socio-economic predictors of adolescent marriage and maternity in West and Central Africa between 1986 and 2017
Source: J Glob Health. 2021 Aug 10;11:13002. doi: 10.7189/jogh.11.13002 (PMC8397280; doi:10.7189/jogh.11.13002)
Supplement: Online Supplementary Document [file jogh-11-13002-s001.pdf]

Table S1: Overview of available data by country and phase

	phase							
country	1	2	3	4	5	6	7	Total
Benin	0	0	5,491	6,219	17,794	16,599	16,348	62,451
Burkina Faso	0	6,354	6,445	12,477	8,159	17,087	8,111	58,633
Cameroon	0	3,871	5,501	15,723	9,408	15,426	10,447	60,376
Central African Republic	0	0	5,884	16,964	15,192	12,507	0	50,547
Chad	0	0	7,454	11,990	0	18,088	17,719	55,251
Congo	0	0	0	0	19,464	10,819	11,841	42,124
Congo, Dem.Rep.	0	0	0	12,409	9,995	32,062	0	54,466
Cote d'Ivoire	0	0	11,139	12,369	23,388	10,060	12,463	69,419
Equatorial Guinea	0	0	0	4,241	0	0	0	4,241
Gabon	0	0	0	6,183	0	8,422	0	14,605
Gambia	0	0	0	5,973	10,252	25,371	0	41,596
Ghana	0	0	4,562	10,534	11,156	16,314	14,546	57,112
Guinea	0	0	0	6,753	7,954	9,142	10,245	34,094
Guinea Bissau	0	0	0	8,206	9,904	0	10,744	28,854
Liberia	5,239	0	0	0	11,489	13,178	4,290	34,196
Mali	3,200	0	9,704	12,849	43,429	10,424	27,196	106,802
Mauritania	0	0	0	0	13,416	13,657	15,133	42,206
Niger	0	6,503	7,577	5,664	9,223	11,160	0	40,127
Nigeria	0	8,781	0	17,430	60,477	45,292	44,210	176,190
Sao Tome and Principe	0	0	0	0	2,615	0	3,101	5,716
Senegal	0	6,310	8,593	11,886	40,698	24,324	52,994	144,805
Sierra Leone	0	0	0	4,905	16,631	16,658	26,507	64,701
Togo	3,360	0	8,569	4,674	6,210	16,496	4,674	43,983
Total	11,799	31,819	80,919	187,449	346,854	343,086	290,569	1,292,495
